# Endothelial Migration and Regeneration after Penetrating Trauma Injury in a Deep Anterior Lamellar Keratoplasty Graft: Case Presentation and Literature Overview

**DOI:** 10.3390/jcm13051424

**Published:** 2024-02-29

**Authors:** Luca Pagano, Alfredo Borgia, Fadi Alfaqawi, Aruni Makuloluwa, Giulia Coco, Giuseppe Giannaccare, Marco Messina, Vito Romano, Kunal Gadhvi

**Affiliations:** 1 Department of Corneal Diseases, St. Paul’s Eye Unit, Royal Liverpool University Hospital, Liverpool L7 8YE, UK; luca.pagano@humanitas.it (L.P.); fadi.alfaqawi@nhs.net (F.A.); aruni.makuloluwa@rlbuht.nhs.uk (A.M.); vito.romano@unibs.it (V.R.); kunal.gadhvi@liverpoolft.nhs.uk (K.G.); 2 IRCCS Humanitas Research Hospital, Via Manzoni 56, 20089 Rozzano, Italy; 3 Eye Unit, Humanitas-Gradenigo Hospital, 10153 Turin, Italy; 4 Department of Clinical Science and Translational Medicine, University of Rome Tor Vergata, 00133 Rome, Italy; giulia.coco@uniroma2.it; 5 Eye Clinic, Department of Surgical Sciences, University of Cagliari, 09124 Cagliari, Italy; giuseppe.giannaccare@unica.it; 6 Department of Biomedical and Surgical Sciences, Section of Ophthalmology, University of Perugia, S. Maria della Misericordia Hospital, 61029 Perugia, Italy; marco.messina@ospedale.perugia.it; 7 Department of Medical and Surgical Specialties, Radiological Sciences, and Public Health, Ophthalmology Clinic, University of Brescia, 25121 Brescia, Italy; 8 Department of Eye and Vision Science, Institute of Life Course and Medical Sciences, University of Liverpool, Liverpool L69 3BX, UK

**Keywords:** DALK, corneal endothelium, ocular trauma, endothelial migration, Descemet membrane

## Abstract

**Background**: Traumatic injuries in eyes previously treated with Deep Anterior Lamellar Keratoplasty (DALK) can lead to ruptures in the Descemet Membrane (DM) and damage to the corneal endothelium, a crucial layer for maintaining corneal clarity. Due to cell cycle constraints, the human corneal endothelium cannot proliferate; instead, it compensates for injury through cell enlargement and migration from adjacent areas. **Methods**: This study examines a notable case of corneal endothelial cell migration following a penetrating eye injury in a patient previously treated with DALK for keratoconus, supplemented by a review of relevant literature to contextualize the regenerative response. **Results**: A 39-year-old male with a history of DALK suffered a traumatic eye injury, resulting in damage to the Descemet Membrane and loss of the crystalline lens. After primary repair and considerations for further surgery, the patient’s cornea cleared remarkably, with an improved visual acuity. This demonstrates the DM’s potential for self-repair through endothelial cell migration. **Conclusions**: The outcomes suggest that delaying corneal transplant surgery for up to 3 months following Descemet Membrane injury due to ocular trauma could be advantageous. Allowing time for natural healing processes might eliminate the need for further invasive surgeries, thereby improving patient recovery outcomes.

## 1. Introduction

Traumatic wound dehiscence (TWD) is a complication of both penetrating keratoplasty (PK) and lamellar keratoplasty that can lead to subsequent graft failure [1]. Blunt trauma is the most common cause of TWD, with the graft–host junction (GHJ) being the primary site of globe rupture [1]. This weakness is attributed to factors such as the lower tensile strength of corneal scars and delayed wound healing due to steroid use. Lamellar keratoplasty is believed to offer better globe integrity and a stronger GHJ, as well as faster healing and better biomechanical properties compared to PK [2].

This article describes a case of potential endothelial cell migration following penetrating ocular damage on a prior deep anterior lamellar keratoplasty (DALK) with GHJ dehiscence and a break in the Descemet membrane (DM). Additionally, we provide an overview of the literature on the case series reported on this topic.

## 2. Case Description

**Figure 1:**
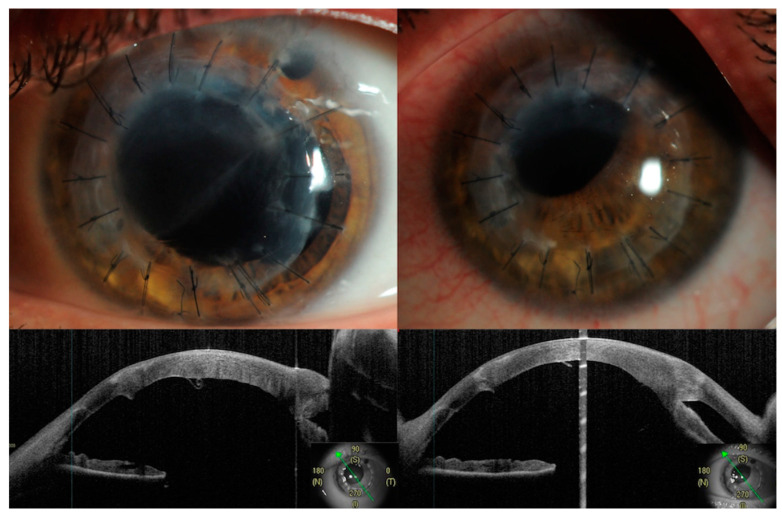
Top row: anterior segment photo showing 1 week (**left**) and 6 months (**right**) post primary surgical repair. On the left, notice the corneal oedema present for the superior 60% of the cornea with a clear line demarcating the break of the Descemet (from 2 to 7 o’clock). In the top right, notice the restoration of the cornea clarity. Bottom row: anterior segment OCT scan at 135° showing a break in the Descemet with a roll configuration (**left**) and the same line in a scan 6 months after (**right**), showing a small Descemet detachment with a resolution of the roll configuration. The green arrow represents the orientation of the anterior segment OCT acquisition.

A 39-year-old male had a history of bilateral keratoconus and had undergone DALK surgery on his left eye one year before. He sustained a left eye blunt trauma after being poked by his son’s finger while playing, and the trauma resulted in corneal graft wound dehiscence and extrusion of the crystalline lens. At presentation, the patient’s visual acuity was equal to hand movement, and upon slit lamp examination, a haziness in the corneal transplant with dehiscence in the GHJ was observed between 8 and 1 o’clock, along with a break in the DM and iris tissue damage. The fundus was not visible, but the retina was found to be attached on gentle B-ultrasound scan. Anterior segment optical coherence tomography (AS-OCT) showed corneal oedema in the superior 60% of the cornea and a demarcation line was visible from 2 to 7 o’clock, which corresponded to the Descemet’s break (Figure 1).

**Figure 2:**
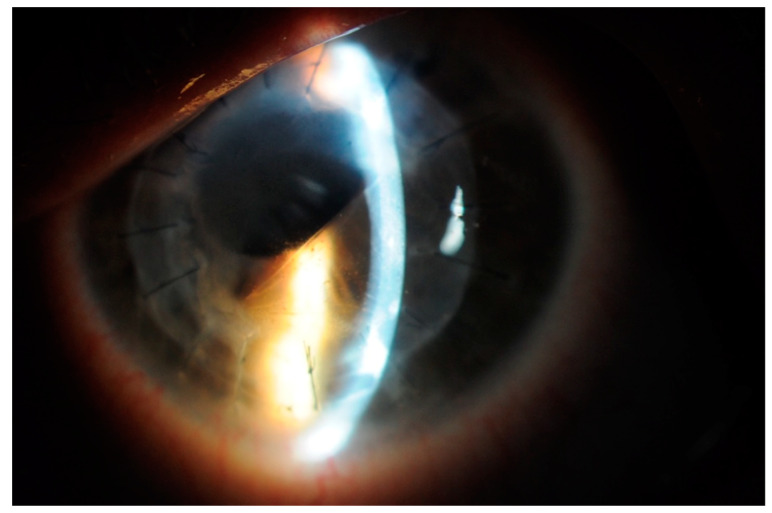
Anterior segment photo showing detail 6 months after. Notice the corrugation of the rolled Descemet (inferiorly) and the clarity of the cornea in the superior half.

The patient underwent primary repair on the same day, which involved an anterior vitrectomy and the use of interrupted 10/0 nylon sutures to secure the wound. Early postoperative evaluations 7 and 10 days later showed that the superior hemicornea appeared edematous with no wound leak and a normal intraocular pressure, and the patient the patient was aphakic. Two months after the primary surgical repair, the patient was scheduled for a left eye pars plana vitrectomy combined with penetrating keratoplasty and a scleral fixated intraocular lens implant. However, 7 weeks after the primary traumatic repair, his corneal oedema decreased and his BCVA slightly improved to 6/24; therefore, it was decided to postpone the new transplant and schedule a follow-up appointment. During subsequent visits, a progressive improvement in visual acuity and clearing of the cornea was observed. Six months after the primary surgical repair, the BCVA was 6/7.5, the cornea appeared completely clear, and the DM appeared unrolled on AS-OCT scans (Figure 1 and Figure 2). The patient was offered a scleral fixated lens implant, but up to now, he has preferred to wait for the surgery for personal reasons, stating that he was happy with his vision.

## 3. Discussion

### 3.1. DALK Advantages

Wound dehiscence can be a complication post keratoplasty and can be divided into traumatic and non-traumatic (spontaneous, post suture removal or microbial keratitis) causes [1]. These patients carry a lifelong risk of wound dehiscence at the graft–host junction secondary to trauma [1,2]. This is thought to be due to the weakened corneal structure due to sub-optimal wound healing and remodeling post keratoplasty [1]. Deep anterior lamellar keratoplasty has several advantages over penetrating keratoplasty, including lower rates of endothelial rejection, reduced intraoperative and postoperative complications, such as iatrogenic cataracts, endophthalmitis and expulsive hemorrhages, and quicker suture removal [2,3,4]. In addition to this, DALK is thought to maintain a superior globe integrity due to preservation of the host Descemet’s membrane, and also has been partly attributed to faster tapering of steroid drops post surgery that leads to quicker wound healing and a stronger graft–host junction. Acar et al. studied corneal hysteresis and the corneal resistance factor in patients who had no previous ocular surgery and in those who had PK and DALK for keratoconus [5]. They showed that these values are significantly lower in patients who have had PK compared to both the control and DALK groups, whereas there were no significant differences between DALK and the control groups.

### 3.2. TWD and DALK

The prevalence of TWD varies between studies due to the random nature of trauma; one study conducted in Saudi Arabia reported similar incidences of 1.9% and 1.7% in PK and lamellar keratoplasty (LKP) groups, respectively [2]. TWD is more likely in male patients who are more inclined to engage in high-risk activities and professions and violence [6]. It is also more prevalent in younger patients [4,6]. Studies have shown blunt trauma to be the most frequent cause of TWD following PK and lamellar keratoplasty [4,7]. Other causes are sports-related accidents, road traffic accidents, assault and falls [2].

The predominant location of globe rupture following keratoplasty is the graft–host junction [4,7]. Al-Othman et al. showed that there were no differences in the extent of trauma (as defined by the number of corneal quadrants involved) between PK and LKP groups, and that there were no associations with the suturing technique (the majority had interrupted sutures), the presence or absence of sutures at the time of TWD, the use of topical steroids at the time of TWD or the recipient trephination size between the two groups [2]. Another study by Goweida et al. also showed that there was no difference in the extent of globe rupture between PK and DALK groups [4].

The study by Al-Othman showed that the PK group had more lens involvement (52.7%) compared with LKP group (23.3%), but no difference in the posterior segment involvement between the two groups [2]. In this study, there were no graft failures, which the authors attributed to the timely management of the globe rupture and the young age of patients, who may have high endothelial cell counts at the time of trauma. In contrast, a study by Goweida et al. reported 50% of the patients required repeat keratoplasty due to graft failure (9/16 PK and 1/4 LKP eyes) approximately 1 year after trauma [4]. This may be related to endothelial cell injury and corneal decompensation from trauma or subsequent surgery. Moreover, the initial indication for keratoplasty in this study included corneal scarring post infection (bacterial and viral), pseudophakic bullous keratopathy and congenital hereditary endothelial dystrophy, in addition to keratoconus.

Interestingly, patients who underwent manual dissection in the LKP group were found to have a greater extent of TWD compared to those who underwent the big bubble technique [2]. We could speculate that this could be explained as the plane obtained through manual dissection is more irregular compared to the one obtained with a big bubble, providing a weaker adhesion. Additionally, it is more superficial, and therefore the area of contact between the donor cornea and the recipient in the periphery is less. In contrast, manually dissection had no lens involvement, whereas 44% of eyes treated with the big bubble technique had lens involvement during trauma, possibly because the additional residual stroma in the manually dissected patients works as an additional protective layer. There were no differences in posterior segment involvement nor the final visual acuity outcome at the last visit post-trauma repair between the manual dissection and big bubble techniques.

The visual outcomes 6 months after wound dehiscence secondary to traumatic and non-traumatic causes were compared by Stevenson et al., who reported the median best correct visual acuity (BCVA) of the traumatic patients to be worse (LogMAR 1.00) compared to the non-traumatic patients (logMAR 0.78), although this did not reach statistical significance [1]. They reported better visual acuities in patients who underwent DALK compared to PK; however, this did not reach statistical difference after adjusting for keratoconus. Al-Othman et al. reported better outcomes post TWD globe repair and also showed no difference in the final visual acuity between PK and LKP groups, with 76% and 73% having VA between 20/20 and 20/60, respectively [2].

### 3.3. Endothelial Cell Migration and Regeneration

TWD in previous DALK could result in a rupture of the recipient DM and corneal endothelium. The adult human corneal endothelium consists of a monolayer of cells arranged in a regular hexagonal array with a cell density of 2000–2500 cells/mm^2^ and functions in maintaining corneal hydration and thus transparency [8]. The DM is a basement membrane synthesized by endothelial cells from prenatal life and thickens with age [9]. The DM helps to maintain the endothelial phenotype [9]. When there is loss or damage to the corneal endothelium, the cells are unable to proliferate as they are locked in the G1 phase of the cell cycle [8]. Instead, the cells increase in size and migrate from the neighboring healthy cells. When the density of cells falls below 500 cells/mm^2^, the human corneal endothelium is not able to regain its homeostasis function and regulate corneal stromal hydration and therefore transparency. The current gold standard for such cases is a corneal graft to replace the injured endothelium.

Since the corneal endothelial layer is relatively difficult to access, there are very limited studies on its healing properties. The process of wound healing in the corneal endothelium has some unique characteristics. In many other tissues, wound healing primarily involves cell proliferation as the main mechanism to reduce and reshape the wound area. However, corneal endothelial cells, particularly in humans, have very slow rates of cell proliferation [10]. As for cell division, some data suggest that it remains very limited during the healing process [11]. It is commonly believed that the corneal endothelium primarily closes the wound gap through cell migration and increased cell spreading [12,13]. Some studies on rabbit corneas seem to agree that cell migration from peripheral endothelial cells is the most likely mechanism responsible for corneal clearing after severe chemical injury and, similarly, corneal oedema caused by iatrogenic and intentional DM avulsion or DMEK graft detachment that resolved spontaneously [14,15,16]. The main signaling pathways involved in corneal endothelial wound healing are the RhoA/ROCK, PI3K/Akt, Wnt and TGF-*β* pathways [17].

### 3.4. Surgical Techniques That Require Endothelial Cell Migration

Experimental surgical techniques such as Descemet’s stripping only (DSO), Descemetorhexis without endothelial keratoplasty (DWEK), and acellular DM transplantation are based on the ability of the patient’s own endothelial cells to migrate and repair the wound [17]. DWEK, which involves a planned central Descemetorhexis without graft implantation in patients with Fuchs endothelial corneal dystrophy, has been associated with variable degrees of success [14,15]. The success of Descemetorhexis has been linked to the patient’s age, with younger patients being more likely to have successful results [18]. A topical ROCK inhibitor has been shown to reduce the corneal clearance time, improve the central endothelial cell density and improve the overall cell architecture when used post DWEK/DSO [17]. It has been postulated that the ROCK inhibitor has a role in endothelial cell proliferation in addition to endothelial cell migration. Several mechanisms for peripheral endothelium regeneration of the central denuded stroma have been proposed, in addition to the well-established migration of adjacent cells [19]. However, none of these have been replicated in vivo in humans. Corneal endothelial stem cells with the ability to proliferate have been identified in the peripheral cornea, but there is still some uncertainty regarding their function in vivo [20].

An ex vivo study on human corneas showed that an intact DM was important for more rapid endothelial cell migration and resulted in greater cell density and hexagonality compared to those without an intact DM [8]. A rabbit study also showed that the presence of an intact DM allowed endothelial cell migration by undergoing a transient fibrotic endothelial–mesenchymal transition that was reversed back to the endothelial phenotype on day 14. This was in contrast to the rabbit corneas without an intact DM, where the cell maintained a fibroblastic phenotype on day 14 [9].

### 3.5. Current Study

The current study presents a rare case of possible endothelial cell migration and regeneration following a penetrating eye trauma with GHJ dehiscence and a break in the DM. The occurrence of this clinical evolution could depend on the low patient’s age, a possible spontaneous DM unfoldment and a healthy and high-density corneal endothelium. An ex vivo study showed that endothelial cell migration was rapid and complete over intact DMs in donor corneas younger than 50 years compared to older corneas [8]. The authors suggested that younger donor corneas may be able to retain the potential to migrate and even proliferate by maintaining and reactivating signaling pathways that may not be present in older corneas. Although corneal oedema following a DM and endothelial damage significantly reduces visual performance, waiting up to three months before proceeding with an endothelial keratoplasty, especially in young patients, could be wise in the presence of corneal oedema due to DM damage or detachment. Studies have shown that repopulation of a bare corneal stromal and gaining corneal clearance can occur between 3 and 6 months post-operatively [17].

Chaniyara et al. presented a method of rescuing a similar break in the DM of a DALK patient following full thickness wound dehiscence [21]. They presented a 22-year-old male who had inferior 180° wound dehiscence following blunt trauma who had edematous donor tissue separated from the host DM which was torn in the paracentral area, with interface fluid on an OCT scan. They undertook globe repair with Descemetopexy with air and closure of the dehiscence with 10-0 nylon sutures. The BCVA improved from hand movements to 20/40 six months after globe repair.

### 3.6. Limitations

The study presented here has several limitations that need to be acknowledged. Firstly, we were unable to determine the exact cause of the spontaneous resolution of corneal oedema. The low patient age along with the possibility of spontaneous DM unfoldment can be considered contributing factors, but further studies are needed to confirm these assumptions. Secondly, the study did not include a specular microscopy examination to determine the endothelial cell count before and after the treatment. This means that we cannot accurately assess the extent of the possible endothelial cell migration and regeneration that occurred in this case. Finally, the study was based on a single case and therefore, the results cannot be considered representative of the general population. Further studies involving larger patient populations are needed to validate the findings of this study and to establish generalizable conclusions.

## 4. Conclusions

In conclusion, this study presents a rare case of spontaneous cornea clearing following a penetrating eye trauma with a GHJ and a break in the DM due to possible endothelial cell migration and regeneration, suggesting the need for observation before proceeding with repeated keratoplasty in similar cases. The limitations of the study highlight the need for further investigation to fully understand the mechanisms and conditions under which these events occur.

## Data Availability

No new data were created or analyzed in this study. Data sharing is not applicable to this article.

## References

[1] Stevenson L.J., Abell R.G., McGuinness M.B., Vajpayee R.B. (2019). Comparative Evaluation of Clinical Characteristics And Visual Outcomes Of Traumatic And Non-Traumatic Graft Dehiscence Following Corneal Transplantation Surgery. Clin. Ophthalmol..

[2] Al-Othman A.Y., AlDhaheri H.S., Ahmad K., Al-Swailem S.A. (2021). Comparison of the Characteristics and Outcomes of Traumatic Wound Dehiscence after Penetrating or Lamellar Keratoplasty for Keratoconus. Eur. J. Ophthalmol..

[3] Reinhart W.J., Musch D.C., Jacobs D.S., Lee W.B., Kaufman S.C., Shtein R.M. (2011). Deep Anterior Lamellar Keratoplasty as an Alternative to Penetrating Keratoplasty: A Report by the American Academy of Ophthalmology. Ophthalmology.

[4] Goweida M.B., Helaly H.A., Ghaith A.A. (2015). Traumatic Wound Dehiscence after Keratoplasty: Characteristics, Risk Factors, and Visual Outcome. J. Ophthalmol..

[5] Acar B.T., Akdemir M.O., Acar S. (2013). Corneal Biomechanical Properties in Eyes with No Previous Surgery, with Previous Penetrating Keratoplasty and with Deep Anterior Lamellar Keratoplasty. Jpn. J. Ophthalmol..

[6] Meyer J.J., McGhee C.N.J. (2016). Incidence, Severity and Outcomes of Traumatic Wound Dehiscence Following Penetrating and Deep Anterior Lamellar Keratoplasty. Br. J. Ophthalmol..

[7] Kawashima M., Kawakita T., Shimmura S., Tsubota K., Shimazaki J. (2009). Characteristics of Traumatic Globe Rupture after Keratoplasty. Ophthalmology.

[8] Soh Y.Q., Peh G., George B.L., Seah X.Y., Primalani N.K., Adnan K., Mehta J.S. (2016). Predicative Factors for Corneal Endothelial Cell Migration. Investig. Ophthalmol. Vis. Sci..

[9] Chen J., Li Z., Zhang L., Ou S., Wang Y., He X., Zou D., Jia C., Hu Q., Yang S. (2017). Descemet’s Membrane Supports Corneal Endothelial Cell Regeneration in Rabbits. Sci. Rep..

[10] Mimura T., Yamagami S., Amano S. (2013). Corneal Endothelial Regeneration and Tissue Engineering. Prog. Retin. Eye Res..

[11] Jeong G.L., Kay E.D.P. (2006). FGF-2-Induced Wound Healing in Corneal Endothelial Cells Requires Cdc42 Activation and Rho Inactivation through the Phosphatidylinositol 3-Kinase Pathway. Investig. Ophthalmol. Vis. Sci..

[12] Joyce N.C., Meklir D., Neufeld A.H. (1990). In Vitro Pharmacologic Separation of Corneal Endothelial Migration and Spreading Responses. Investig. Ophthalmol. Vis. Sci..

[13] Gordon S.R. (1994). Cytological and Immunocytochemical Approaches to the Study of Corneal Endothelial Wound Repair. Prog. Histochem. Cytochem..

[14] Braunstein R.E., Airiani S., Chang M.A., Odrich M.G. (2003). Corneal Edema Resolution after “Descemetorhexis”. J. Cataract. Refract. Surg..

[15] Watson S.L., Abiad G., Coroneo M.T. (2006). Spontaneous Resolution of Corneal Oedema Following Descemet’s Detachment. Clin. Exp. Ophthalmol..

[16] Choi S.O., Jeon H.S., Hyon J.Y., Oh Y.J., Wee W.R., Chung T.Y., Shin Y.J., Kim J.W. (2015). Recovery of Corneal Endothelial Cells from Periphery after Injury. PLoS ONE.

[17] Vercammen H., Miron A., Oellerich S., Melles G.R.J., Ní Dhubhghaill S., Koppen C., Van Den Bogerd B.E.R.T. (2022). Corneal Endothelial Wound Healing: Understanding the Regenerative Capacity of the Innermost Layer of the Cornea. Transl. Res..

[18] Moloney G., Chan U.T., Hamilton A., Zahidin A.M., Grigg J.R., Devasahayam R.N. (2015). Descemetorhexis for Fuchs’ Dystrophy. Can. J. Ophthalmol..

[19] Joyce N.C. (2012). Proliferative Capacity of Corneal Endothelial Cells. Exp. Eye Res..

[20] Yokoo S., Yamagami S., Yanagi Y., Uchida S., Mimura T., Usui T., Amano S. (2005). Human Corneal Endothelial Cell Precursors Isolated by Sphere-Forming Assay. Investig. Ophthalmol. Vis. Sci..

[21] Chaniyara M.H., Bafna R., Urkude J., Sharma N. (2017). Rescuing the Host Descemet’s Membrane in Full-Thickness Traumatic Wound Dehiscence in Deep Anterior Lamellar Keratoplasty: Intraoperative Optical Coherence Tomography (IOCT)-Guided Technique. Case Rep..

